# Stigmasterol-Based EGCG Liposomes Reduce Nε-(carboxymethyl)lysine (CML) and Nε-(carboxyethyl)lysine (CEL) in a Model System and Cookies

**DOI:** 10.3390/foods15111997

**Published:** 2026-06-03

**Authors:** Xinyu Liu, Wei Quan, Xufeng Wang, Yunhui Cheng, Ye Jiao

**Affiliations:** 1 School of Food Science and Bioengineering, Changsha University of Science & Technology, Changsha 410114, China; liuxinyu1007@stu.csust.edu.cn (X.L.); xufengw@csust.edu.cn (X.W.); cyh@csust.edu.cn (Y.C.); 2 College of Food Science and Technology, Hunan Agricultural University, Changsha 410128, China; reus_quan@hunau.edu.cn

**Keywords:** (–)-epigallocatechin gallate, stigmasterol, liposomes, advanced glycation end products, cookies

## Abstract

(–)-Epigallocatechin gallate (EGCG) is a promising inhibitor for the formation of advanced glycation end products. However, its instability limits its application in complex food systems. In this study, EGCG was encapsulated into liposomes prepared using stigmasterol as a cholesterol substitute. The optimal formulation (mass ratio of lecithin: stigmasterol: EGCG = 10:5:1) achieved a high encapsulation efficiency of 92.86% and a particle size of 239.87 nm. Stigmasterol-based EGCG liposomes (ESLs) significantly enhanced the stability of EGCG at 100 °C across the pH range of 5.0–8.0, and also notably improved its antioxidant activity. Moreover, ESL increased the trapping efficiency of EGCG against glyoxal and methylglyoxal under thermal conditions. Consequently, ESLs exhibited a stronger inhibitory effect on Nε-carboxymethyllysine (CML) and Nε-carboxyethyllysine (CEL) formation than that of free EGCG both in the chemical model system and in cookies. When applied in cookies at an optimal concentration of 0.05%, ESLs reduced CML and CEL by 45.8% and 47.0% respectively, with only minor impacts on texture and color. These results indicate that encapsulation of EGCG into stigmasterol-based liposomes effectively protects it, thus maintaining its stability and inhibitory activity in real food matrices.

## 1. Introduction

Advanced glycation end products (AGEs) are stable products formed through non-enzymatic reactions between reducing sugars and free amino groups of proteins, lipids, or nucleic acids [1], including oxidation, dehydration, and molecular rearrangement [2]. Due to their stability and resistance to degradation in the body, AGEs can continuously accumulate in the human circulatory system, ultimately leading to tissue damage and the development of diseases [3]. Research has shown that the intake of AGEs is associated with diabetes and its complications, chronic renal failure, Alzheimer’s disease, cardiovascular diseases, dyslipidemia, and accelerated skin aging [4]. Therefore, reducing the formation of AGEs in food is crucial for human health. Among AGEs, Nε-(carboxymethyl)lysine (CML) and Nε-(carboxyethyl)lysine (CEL) exhibit stability and universality in food, and are thus frequently used as the primary target markers for measuring AGEs in food products [5].

The formation of CML and CEL is associated with the oxidative generation of reactive carbonyl intermediates, such as glyoxal (GO) and methylglyoxal (MGO). (–)-Epigallocatechin gallate (EGCG), a major green tea polyphenol, intervenes at two stages of this pathway: it scavenges reactive oxygen species (ROS) to suppress the formation of GO or MGO [6], and it directly traps any remaining intermediates to prevent the accumulation of AGEs [7]. However, EGCG shows instability under thermal processing conditions, which limits its application in thermally processed foods [8]. Moreover, its poor lipid solubility renders it unsuitable for use in high-fat foods [9]. To address these practical challenges in the application of active compounds such as EGCG, a large amount of research has focused on designing delivery carrier systems for efficient and protective encapsulation. Among the available options, liposomes are a promising choice. They can not only enhance the thermal stability of the encapsulated active compounds during thermal processing but also improve their functional performance in high-fat food matrices while preserving the intrinsic molecular structure of the active compounds [10,11].

Liposomes are self-assembling nanostructures composed of a phospholipid bilayer, in which the hydrophilic head groups face the external and internal aqueous environments, while the region between the two leaflets consists of hydrophobic tail [12,13]. Liposomal encapsulation has been effectively applied to deliver diverse bioactive polyphenols, such as olive polyphenols [14], p-Coumaric acid [15], and *Lycium barbarum* leaf polyphenols [16]. All of these encapsulated polyphenols exhibit greater antioxidant activity than their corresponding free compounds. Meanwhile, it has been demonstrated that the thermal stability of white tea extract encapsulated in liposomes is better than that of the unencapsulated extract [17]. Collectively, these findings support the potential of liposomal delivery to enhance the stability, bioactivity, and functional performance of EGCG.

Cholesterol is well-known to contribute to the stability of phospholipid bilayers, and is routinely incorporated into liposomal formulations [18]. Within the bilayer, cholesterol promotes tight packing of fatty acyl chains and stabilizes a liquid-ordered phase [19]. However, it is associated with an increased risk of developing cardiovascular disease [20]. In contrast, stigmasterol, a common phytosterol structurally analogous to cholesterol (as shown in Figure S1) [21], does not affect the development of cardiovascular disease [22]. Meanwhile, it exhibits multifaceted bioactivity, including antitumor effects, induction of tumor cell apoptosis, and modulation of gut microbiota composition [23]. Furthermore, it has been successfully applied to deliver unstable bioactive compounds, such as antihypertensive oligopeptides [24], luteolin [25], and astaxanthin [26]. Therefore, stigmasterol is a physiologically safer and functionally equivalent alternative to cholesterol for liposome design.

In this study, liposomes loaded with EGCG were formulated, and stigmasterol was evaluated as a potential cholesterol substitute within the liposomal bilayer. The stability, antioxidant activity, and reactive dicarbonyl compound trapping capacity of EGCG were assessed in both its free form and liposomal encapsulated state. Furthermore, the inhibitory effect of EGCG-loaded liposomes on AGEs formation was investigated in both a chemical model and a realistic food matrix (cookies), along with a concurrent sensory evaluation of the cookie samples to determine consumer acceptability.

## 2. Materials and Methods

### 2.1. Chemicals

EGCG (≥98%) was purchased from Spring & Autumn Biological Engineering Co., Ltd. (Nanjing, China). CML was obtained from Yuanye Bio-Technology Co., Ltd. (Shanghai, China). Lecithin from soybean (biotechnology grade, meeting the quality standards for soya lecithin in the Chinese Pharmacopoeia [27] and European Pharmacopoeia [28]: phosphatidylcholine (PC) ≥ 45.0%, phosphatidylethanolamine (PE) ≤ 30.0%, total PC + PE ≥ 70.0%), cholesterol (>95%), stigmasterol (>90%), Triton X-100 (97%), GO (40% aqueous solution), MGO (40% aqueous solution), oleic acid (85%), lysine (98%) and CEL were all supplied by Macklin Biochemical Co., Ltd. (Shanghai, China). Anhydrous ethanol (AR), glucose (AR), o-phenylenediamine (CP) and 2,3-hexanedione (94%) were acquired from Sinopharm Chemical Reagent Co., Ltd. (Shanghai, China). Acetonitrile (HPLC) was sourced from Merck & Co., Inc. (Kenilworth, NJ, USA). Wheat flour, sucrose, skimmed milk powder, salt, baking soda, baking shortening, high-fructose corn syrup, and ammonium bicarbonate were all bought from local supermarkets.

### 2.2. Preparation of EGCG-Loaded Liposomes

Liposomes were prepared using the thin-film dispersion method, adapted from Chen (2019) [29]. Briefly, for the formulation 10:5:1, 100 mg lecithin, 50 mg stigmasterol, and 10 mg EGCG were dissolved in 100 mL of ethanol at predetermined mass ratios and transferred to a round-bottomed flask. For other formulations, lecithin, EGCG, and either cholesterol or stigmasterol were added in appropriate ratios. The organic solvent was removed by rotary evaporation at 50 °C, yielding a lipid film on the inner surface of the flask. The film was then hydrated with 150 mL of phosphate buffer (10 mmol/L, pH 7.0) at 50 °C for 30 min under gentle swirling to ensure complete hydrating of the lipid film. Subsequently, the hydrated dispersion was sonicated in a water bath using an ultrasonic processor (KQ-400KDE, Kunshan Ultrasonic Instruments Co., Ltd., Kunshan, China) operating at 40 kHz and 400 W for 4 min. The resulting EGCG-loaded liposomal suspensions were stored at 4 °C in the dark. Liposomes composed of lecithin and EGCG are denoted by ELs; those composed of lecithin, cholesterol, and EGCG are denoted by ECLs; and those composed of lecithin, stigmasterol, and EGCG are denoted by ESLs.

### 2.3. Determination of Particle Size, Zeta Potential and Encapsulation Efficiency (EE)

The particle size and zeta potential of the EGCG-loaded liposomes were determined by dynamic light scattering using a NanoBrook 90plus Zeta particle size analyzer (Brookhaven Instruments, Brookhaven, NY, USA). Measurements were conducted on samples diluted 50-fold at a constant temperature of 25 °C.

The EE of EGCG-loaded liposomes was assessed by the ultrafiltration–centrifugation method [30]. Briefly, 400 μL of the liposomal suspension was loaded into an ultrafiltration centrifuge tube (molecular weight cutoff: 3 kDa) and centrifuged at 10,000 r/min for 20 min. The filtrate was collected, and the concentration of free EGCG was quantified by high-performance liquid chromatography (HPLC) with ultraviolet detection at 273 nm, using a ShimNex CS C18 column (150 mm × 4.6 mm I.D., 5 μm). In parallel, 500 μL of the same suspension was mixed with an equal volume of Triton X-100, vigorously vortexed to ensure the complete disruption of the bilayer, and diluted to 5 mL with deionized water. The resulting solution was filtered through a 0.22 μm membrane, and the total EGCG content was also determined by HPLC. EE was calculated as

(1) $$\mathrm{EE}\;\left(\%\right)=(1-\frac{\mathrm{free}\;\mathrm{EGCG}}{\mathrm{total}\;\mathrm{EGCG}})\times 100\%$$

### 2.4. Analysis of Stability

The ESL suspension was adjusted to pH 5.0, 6.0, 7.0, and 8.0 using 0.1 mol/L NaOH or HCl. Separately, EGCG was dissolved in phosphate buffer solutions (10 mmol/L, pH 7.0) at the corresponding pH values (5.0, 6.0, 7.0, and 8.0). ESL suspension and EGCG solutions at each pH were then separately transferred into screw-cap sealed reaction vials and subjected to thermal treatment at 100 °C for 30 min. Unheated samples at each pH served as respective controls. After heating, the vials were rapidly cooled in an ice bath to stop the reaction. The extraction and determination of EGCG were performed according to the procedure described in Section 2.3, and the retention rate was calculated using the following equation:

(2) $$\mathrm{Retention}\;\mathrm{rate}\;\left(\%\right)=\frac{\mathrm{EGCG}\;\mathrm{after}\;\mathrm{heat}\;\mathrm{treatment}}{\mathrm{total}\;\mathrm{EGCG}}\times 100\%$$

### 2.5. Evaluation of Antioxidant Capacity

The antioxidant capacity of the samples was evaluated by measuring DPPH and ABTS^+^ radical scavenging activities, ferrous ion (Fe^2+^) chelating capacity, and peroxide value (POV). The DPPH and ABTS^+^ radical scavenging activities of EGCG-loaded liposomes were quantified spectrophotometrically according to the protocols of Rumpf et al. (2023) [31]. Fe^2+^ chelating capacity was determined using the ferrozine-based colorimetric assay described by Paulina et al. (2017) [32]. POV was assessed by iodometric titration following the procedure of Mehta et al. (2015) [33].

### 2.6. Analysis of Intermediates Trapping Capacity

The intermediate trapping capacity was determined according to the method described by Jiao et al. (2019) [34], with minor modifications. In short, reaction mixtures containing equal concentrations (5 mmol/L) of GO and MGO, with varying concentrations of EGCG or ESL, were incubated in citrate–phosphate buffer (pH 7.0) at 80, 100, and 120 °C for 30 min in screw cap-sealed reaction vials. A system without EGCG or ESL served as a control. After thermal incubation, the vials were immersed in an ice-water bath to terminate the reaction. The residual GO and MGO concentrations were then quantified by ultra-performance liquid chromatography coupled with triple-quadrupole mass spectrometry (UPLC-MS/MS, Acquity UPLC TQD, Waters Corporation, Milford, MA, USA) operating in multiple reaction monitoring mode.

### 2.7. Preparation of Chemical Model Systems

The chemical model systems were prepared following a previously reported method [35]. Briefly, 0.1 mol/L glucose and 0.1 mol/L lysine were dissolved in 0.1 mol/L phosphate buffer at different pH values (5.0, 6.0, and 7.0). A 5 mL aliquot of this mixture was transferred into a screw-cap-sealed vial, and then EGCG or ESL was added. The vials were incubated in a preheated oil bath at different temperature (80, 100, and 120 °C) for 30 min. Immediately after incubation, the vials were rapidly cooled in an ice-water bath to quench the reaction.

### 2.8. Preparation of Cookies

Dough was prepared in accordance with the American Association of Cereal Chemists (AACC) Method 10–54 (AACC, 2000) [36]. The formulation consisted of 40.0 g of flour, 16.8 g of sucrose, 16.0 g of baking shortening, 0.5 g of sodium chloride, 0.6 g of sodium bicarbonate, 0.6 g of high-fructose corn syrup, 0.4 g of nonfat dry milk, and 8.8 mL of water. All ingredients were mixed using a Kenwood Chef dough mixer. EGCG or ESL was added to the dough during the final mixing stage and the dough was mixed for an additional 2 min to ensure uniform distribution. Subsequently, the dough was sheeted into circular specimens (55 mm in diameter × 3 mm in thickness) and baked in a convection oven (DEMASHI, Guangdong Demashi Intelligent Kitchen Equipment Co., Ltd., Foshan, Guangdong, China) at 180 °C for 11 min.

### 2.9. Extraction and Quantification of CML and CEL

CML and CEL were extracted and quantified following a previously published approach with appropriate modifications [34]. In the chemical model systems, samples were directly subjected to solid-phase extraction (SPE) before determination. For cookie samples, the extraction process involved defatting with n-hexane, reduction using sodium borohydride, acid hydrolysis with HCl, purification by SPE, and filtration through a 0.22 μm membrane prior to injection. The quantification of CML and CEL was carried out using UPLC-MS/MS coupled with an ACQUITY UPLC C18 column (2.1 mm × 100 mm, 3.5 μm). The mobile phase consisted of 0.1% formic acid in water (solvent A) and acetonitrile (solvent B). The gradient elution program was set as follows: at 0 min, the mobile phase was composed of 95% A; from 0 to 5 min, the proportion of A was decreased to 40%; from 5 to 6 min, it was further reduced to 0%; this condition was maintained from 6 to 7 min; then, from 7 to 8 min, the composition was returned to 95% A, which was held until 10 min. The flow rate was maintained at 0.2 mL/min, and the column temperature was set to 35 °C. Mass spectrometric detection was performed in electrospray ionization positive mode under the following conditions: the capillary voltage was set to 3.55 kV, the source temperature to 150 °C, and the desolvation temperature to 400 °C. The desolvation gas (nitrogen) flow rate was 800 L/h, and the cone gas (nitrogen) flow rate was 50 L/h. Data acquisition and processing were carried out using MassLynx V4.1 software.

### 2.10. Determination of the Color and Texture of Cookies

Cookie texture was determined using a P/2 probe based on Cheng (2014) [37] with modifications. Parameters: pre-test 1.0, test 1.0, post-test 10.0 mm/s; auto trigger 5.0 g. Three penetrations per cookie were averaged, and each sample was measured in triplicate.

The colorimeter was adjusted to the CIE L*a*b* color space and calibrated with a standard white plate. For each cookie, bread, or meatball, L*, a* and b* values were measured at six independent positions using a chromameter.

### 2.11. Statistical Analysis

All experiments were conducted in triplicate, and data are presented as mean ± standard deviation (SD). Statistical significance was assessed using one-way analysis of variance (ANOVA) followed by Tukey’s post hoc test for multiple comparisons, implemented in IBM SPSS Statistics 20.0 (IBM Corp., Armonk, NY, USA). Differences were considered statistically significant at *p* < 0.05.

## 3. Results

### 3.1. Characteristics of EGCG-Load Liposomes

The particle size, polydispersity index (PDI), zeta potential, and EE of EGCG-loaded liposomes with varying compositions are summarized in Table 1. The particle size of ESLs was 239.87 nm, comparable to that of ECLs (234.51 nm). Their PDI values were 0.290 and 0.292, respectively, both below the generally accepted threshold of 0.3 for a monodisperse population [38]. Both formulations exhibited strongly negative zeta potentials (approximately −53 mV), indicating good colloidal stability due to electrostatic repulsion, which helps prevent vesicle aggregation over time [39]. Notably, ESLs and ECLs achieved high EE of 90.91% and 89.83% respectively, which far exceeded the 66.66% obtained with ELs. These findings suggest that stigmasterol can effectively replace cholesterol as a membrane-stabilizing component in EGCG-loaded liposomes. Consistent with this, Hwang et al. (2010) [25] reported that, in an oligopeptide-loaded liposomal system, stigmasterol-based liposomes showed no statistically significant differences from cholesterol-based counterparts in EE, storage stability, pH stability, or oxidative stability. Similarly, Lee et al. (2020) [40] demonstrated that stigmasterol could maintain membrane rigidity and drug loading capacity at levels comparable to cholesterol in liposomal systems. According to Hodzic et al. (2017) [41], the ethyl group and double bond at the C17 position of stigmasterol’s side chain effectively modulates phospholipid bilayer thickness and elasticity.

Based on the experimental results, stigmasterol was selected for further optimization of its molar ratio in the liposomal formulation. As the stigmasterol ratio increased from 10:1:1 to 10:5:1, the particle size progressively increased from 152.06 nm to 224.79 nm, accompanied by a corresponding increase in EE from 89.96% to 94.86%. During this range, the PDI also gradually rose from 0.257 to 0.290, indicating a modest broadening of size distribution while still remaining within the acceptable limit. However, a further increase to 10:7:1 resulted in a rise in particle size to 365.23 nm and a reduction in EE to 85.59%, and the PDI increased further to 0.326, exceeding the 0.3 threshold and confirming the onset of aggregation. These trends align with those reported by Pavlovic et al. (2023) [42], who found that moderate sterol incorporation improved liposomal stability. However, excessive sterol incorporation beyond the optimal concentration caused supersaturation and aggregation of phospholipid molecules, ultimately leading to a lower absolute zeta potential and an unstable state. A similar detrimental effect on size uniformity was observed by Zhao et al. (2015) [43], where increasing β-sitosterol concentration in liposomes progressively raised both particle size and PDI. Moreover, the impact of lecithin content was subsequently investigated by varying the EGCG-to-lecithin ratio. When the mass ratio was increased from 5:5:1 to 20:5:1, both EE and particle size showed an initial increase followed by a decrease. The highest EE and the smallest particle size were achieved at a ratio of 10:5:1. The PDI followed a trend of first decreasing and then increasing with the phospholipid content, indicating that both phospholipid insufficiency and excess compromise the uniformity of the liposomal population. This phenomenon can be attributed to phospholipid insufficiency, which compromises membrane integrity and leads to drug leakage and reduced EE (Zhao et al., 2026) [44]. Conversely, phospholipid excess induces phase separation, thereby reducing the effective drug loading capacity. (Pires et al., 2019) [45]. Based on the highest EE and suitable particle size, the formulation with a mass ratio of lecithin:stigmasterol:EGCG = 10:5:1 was selected for all subsequent experiments.

### 3.2. Stability of ESL

**Figure 1:**
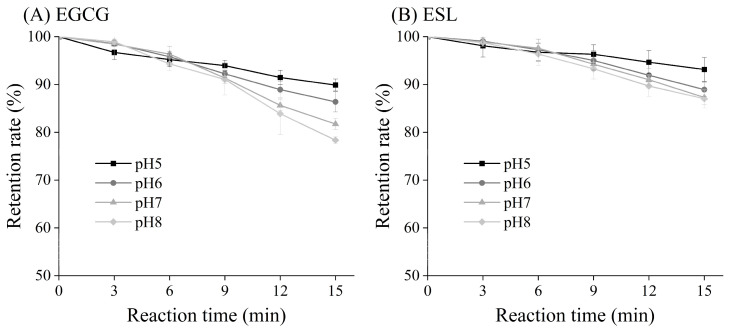
Retention rate of EGCG in the free form (**A**) and in EGCG–stigmasterol–lecithin liposomes (ESLs, (**B**)) after heating at 100 °C at different pH values.

To assess EGCG stability during thermal processing, its retention rate after heat treatment at varying pH levels was determined (Figure 1). Both free EGCG and liposome-encapsulated EGCG showed decreasing retention with increasing heating time. After 15 min of heating, the retention of free EGCG ranged from 78.63% to 89.89%, whereas that of ESL ranged from 87.02% to 93.15%, confirming the protective role of liposomal encapsulation. Retention declined progressively as pH increased. Notably, at pH 8, while free EGCG underwent significant degradation (with a retention rate of only 78.36%), ESLs maintained a high retention rate of 87.02%, indicating their protective capacity under alkaline conditions. EGCG has a pKa_1_ of 7.5, and thus it mainly exists as an undissociated molecule with low electrophilic reactivity at pH 5 Zaabalawi et al. (2022) [46]. At pH 6–7, partial ionization of the hydroxyl groups on the A- and B-rings generates phenoxide anions with increased electron density, thereby enhancing their susceptibility to electrophilic attack by oxygen and metal ions [47]. At pH 8, full ionization of the pyrogallol group in the B-ring initiates rapid autoxidation through semiquinone radical intermediates [48]. Conversely, incorporating EGCG into the liposomal bilayer enables effective physical encapsulation [49], thereby shielding it from oxidants and inhibiting the oxidative degradation pathways described above.

### 3.3. Antioxidative Capacity of ESL

**Figure 2:**
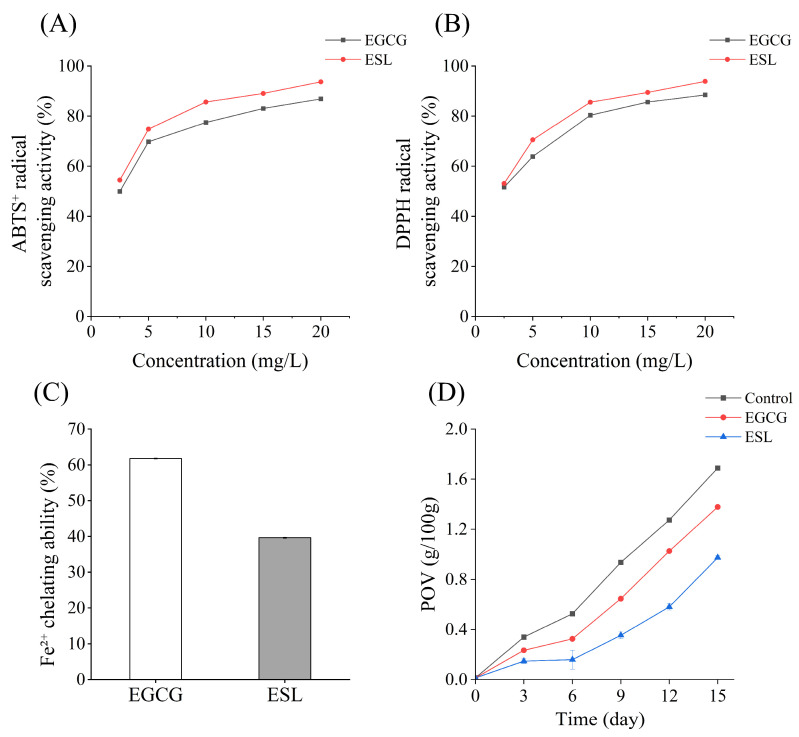
Antioxidant capacity of EGCG–stigmasterol–lecithin liposomes (ESLs) and free EGCG. ESL concentrations are expressed in terms of equivalent EGCG content (*n* = 3). (**A**) ABTS^+^ radical scavenging activity; (**B**) DPPH radical scavenging activity; (**C**) Fe^2+^ chelating activity; (**D**) Peroxide value (POV). The results shown are the outcomes of three independent experiments.

The antioxidant capacity of free EGCG and ESLs was evaluated using ABTS^+^ radical scavenging, DPPH radical scavenging, Fe^2+^ chelating, and POV assays. As depicted in Figure 2A,B, both free EGCG and ESLs exhibited concentration-dependent increases in radical scavenging activity, with ESLs consistently displaying superior efficacy. This enhanced activity is likely attributable to lecithin, the primary phospholipid constituent of ESLs, which possesses certain radical-scavenging capacity [50]. Additionally, it may arise from the protection of the liposomal structure against oxidative degradation of EGCG, thereby preserving its bioactivity [51]. In line with the above observations, Liang et al. (2025) [52] demonstrated that tea polyphenol liposomes exhibited significantly higher ABTS^+^ and DPPH radical scavenging activities than free tea polyphenols.

In contrast, as depicted in Figure 2C, free EGCG exhibited significantly higher Fe^2+^ chelating activity (61.75%) than ESLs (39.65%, *p* < 0.05). The hydroxyl groups on the D-ring of EGCG occupy the primary coordination sphere of metal ions, facilitating formation of a diolate chelate ring. The D-ring serves as the dominant structural determinant for EGCG-metal complexation. Moreover, the hydroxyl groups on the B-ring also engage in weak interactions with metal ions [53]. The reduced Fe^2+^ chelating activity of ESLs is likely due to the phospholipid bilayer, which sterically hinders Fe^2+^ access to critical hydroxyl groups, especially the metal-binding sites on the B- and D-ring [54].

POV is a well-established marker of primary lipid oxidation (Figure 2D). During 15 days of accelerated storage at 60 °C, ESLs had better antioxidant protection than both free EGCG and the control. On day 15, the POV of ESLs was only 0.97 g/100 g, and this value was significantly lower than that of either group throughout the storage period. The marked reduction in POV observed with ESLs can be ascribed to the enhanced stability of EGCG within the liposomal bilayer and the facilitated retention and distribution of the liposomes themselves within the oil phase. This encapsulation facilitates better distribution and retention within the oil phase, thereby enhancing their antioxidant efficacy in lipid matrices [10]. In line with these results, Jahanfar et al. (2020) [55] found that when liposome-encapsulated green tea extract was applied to canola oil, the POV remained consistently lower than that of the group treated with free extract throughout the 32-day storage period.

### 3.4. Reactive Dicarbonyl Compound Trapping Capacity of ESLs

**Figure 3:**
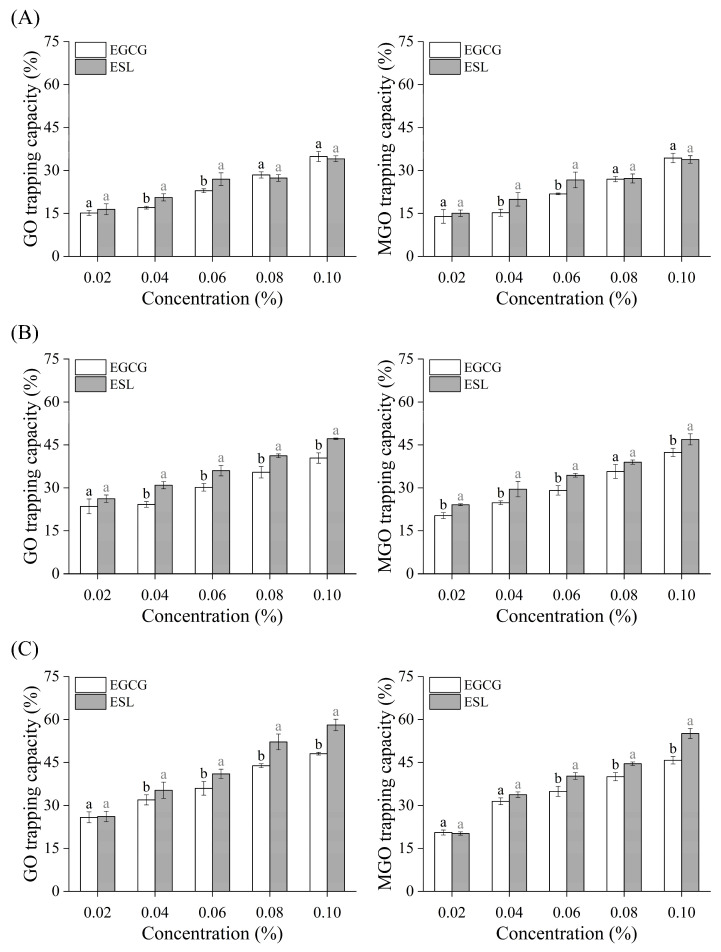
Effects of EGCG and EGCG–stigmasterol–lecithin liposomes (ESLs) on the reduction in GO and MGO in the model system after heating at (**A**) 80 °C, (**B**) 100 °C, and (**C**) 120 °C. ESL concentrations are expressed in terms of equivalent EGCG content. Different letters (*p* < 0.05) indicate significant differences in the trapping capacity of free EGCG and ESLs.

As the addition level of EGCG and ESLs increased, the reduction in GO and MGO rose progressively (Figure 3). At 80 °C, EGCG and ESLs showed comparable efficacy in reducing these dicarbonyls. With rising heating temperature, both EGCG and ESLs exhibited enhanced activity. Notably, ESLs consistently outperformed free EGCG. Specifically, at 120 °C and 0.1% concentration, ESLs reduced GO and MGO by 58.1% and 55.1%, respectively. These values significantly exceeded the reductions achieved by free EGCG (48.3% and 46.7%). The C6 and C8 positions on the A ring of EGCG form mono- or di-adducts with MGO or GO through electrophilic substitution reactions [56]. The superior trapping performance of ESLs likely stems from stigmasterol-mediated membrane stabilization. As demonstrated by Igielska et al. (2025) [57], phytosterol-enriched liposomes exhibit increased membrane density and rigidity above 60 °C, effectively suppressing phospholipid bilayer phase transitions and associated permeability increases, which preserves the bioactivity of the encapsulated core.

### 3.5. Effects of ESLs on CML and CEL Formation in the Chemical Model System

**Figure 4:**
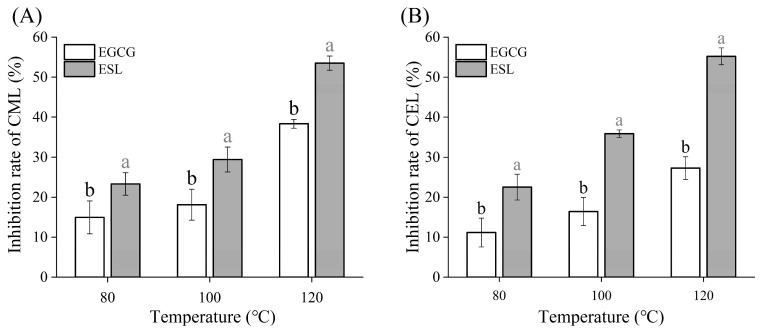
Effects of EGCG and EGCG–stigmasterol–lecithin liposomes (ESLs) on (**A**) CML and (**B**) CEL formation at different temperatures Different letters (*p* < 0.05) indicate significant differences between the EGCG and ESL groups.

The inhibitory effects of ESLs and EGCG on the formation of CML and CEL were first evaluated at different heating temperatures in chemical model systems. As depicted in Figure 4, ESLs demonstrated superior inhibitory effects on both CML and CEL at various temperatures, with efficacies ranging from 1.40 to 1.62 times and 2.01 to 2.19 times that of EGCG, respectively. Furthermore, the inhibitory effects of ESLs on CML and CEL gradually enhanced as the temperature of the model system increased. At 120 °C, ESLs displayed the highest inhibitory activity, with inhibition rates of 53.50% against CML and 55.22% against CEL. The better inhibitory effect of ESLs can be ascribed to the protective effect of liposomes, which stabilizes EGCG, and an improved trapping capacity for GO and MGO under high-temperature conditions, as previously confirmed.

**Figure 5:**
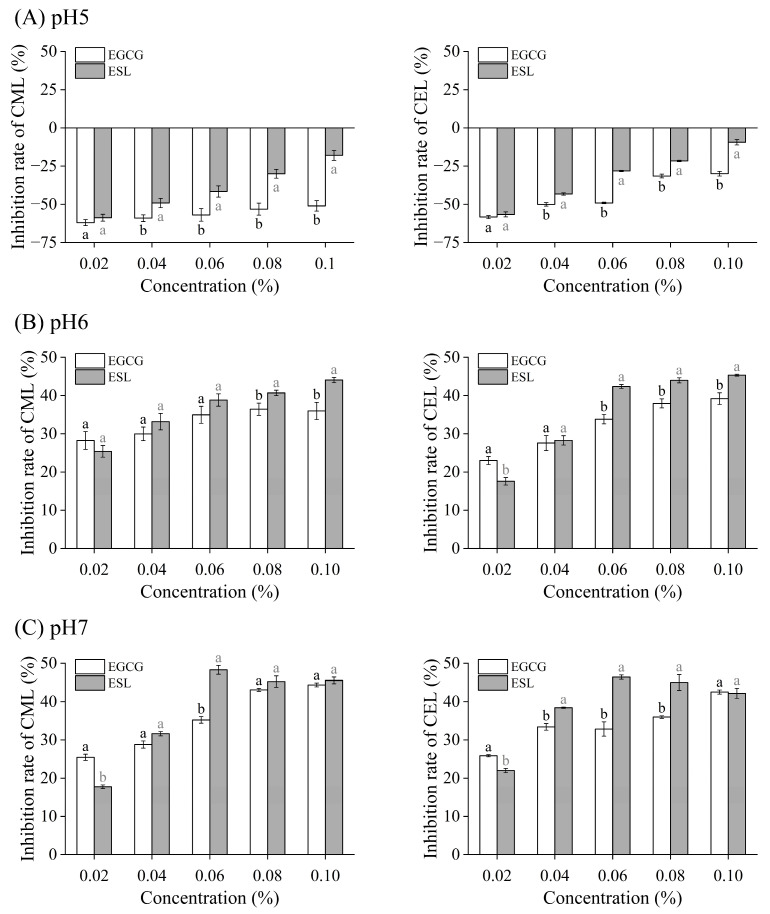
Effects of EGCG and EGCG–stigmasterol–lecithin liposomes (ESLs) on CML and CEL formation at pH values of (**A**) 5, (**B**) 6, and (**C**) 7 after heating at 100 °C. ESL concentrations are expressed in terms of equivalent EGCG content. Different letters (*p* < 0.05) indicate significant differences between the EGCG and ESL groups.

The formation of CML and CEL is significantly influenced by the pH value of the system. Therefore, the effects of ESL on the formation of CML and CEL were further investigated at different pH levels in chemical model systems. As shown in Figure 5B,C, ESLs exhibited significantly stronger inhibition than free EGCG at pH 6 and pH 7. The highest inhibitory activity was observed in the ESL group at pH 7, with inhibition rates of 48.3% for CML and 46.4% for CEL. This may be because ESLs exhibit higher antioxidant activity than EGCG, which is beneficial for inhibiting the formation of CML and CEL, as their formation involves an oxidative process. Additionally, it could be due to the superior intermediate trapping capability of ESLs compared to EGCG at elevated heating temperatures. In contrast, both EGCG and ESLs promoted the formation of CML and CEL at pH 5. Nevertheless, the promoting effect of ESLs was significantly weaker than that of EGCG. According to Jiao et al. (2019) [34], EGCG can undergo autoxidation of its catechol moieties at pH 5, generating hydrogen peroxide that accelerates the formation of CML and CEL. The liposomal encapsulation in ESLs may stabilize EGCG against autoxidation, thereby preserving its inhibitory effect. This observation aligns with prior evidence demonstrating that liposome-based delivery systems can effectively protect EGCG from degradation under various environmental stresses [54].

### 3.6. Effects of ESLs on CML and CEL Formation in Cookies

**Figure 6:**
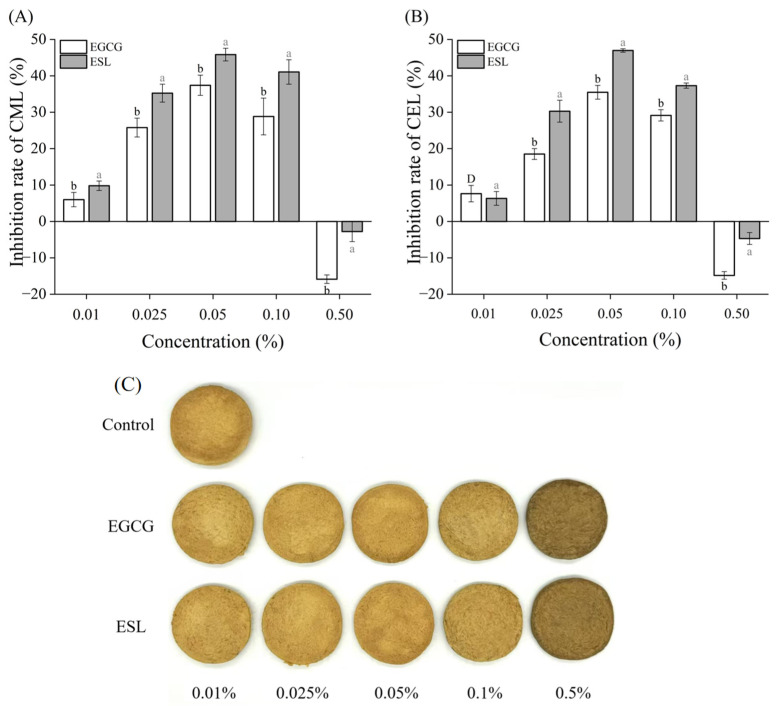
Effects of EGCG and EGCG–stigmasterol–lecithin liposomes (ESLs) on (**A**) CML and (**B**) CEL formation in cookies, and (**C**) photographs of cookies subjected to different treatments. ESL concentrations are expressed in terms of equivalent EGCG content. Different letters (*p* < 0.05) indicate significant differences between the EGCG and ESL groups.

The effects of EGCG and ESL on the formation of CML and CEL in cookies are presented in Figure 6A,B. In the concentration range of 0.01–0.10%, ESLs showed stronger inhibitory effects than EGCG and achieved the highest inhibition rates against CML and CEL, reaching 45.8% and 47.0%, respectively. Although both EGCG and ESL promoted the formation of CML and CEL at an addition level of 0.50%, ESLs exhibited a significantly attenuated promotional effect compared with free EGCG, and this phenomenon was similar to that observed in the chemical model system. Free EGCG demonstrates poor stability under in vitro conditions, undergoing autoxidation to generate hydrogen peroxide and oxidation products [58]. This pro-oxidant behavior probably contributes to the elevated formation of CML and CEL observed at higher addition levels [34]. In comparison, liposomal encapsulation confers substantial protection against thermal and oxidative degradation, and the enhanced antioxidant activity and intermediate-trapping capacity of ESLs collectively contribute to a stronger inhibitory effect against CML and CEL formation. This is supported by Pamunuwa et al. (2023) [59], who encapsulated curcumin and α-tocopherol in liposomes for cookie applications. The liposomes preserved the antioxidant activity during baking, confirming that liposomal encapsulation effectively protects heat-sensitive compounds under thermal processing.

### 3.7. Cookie Texture and Color

In texture profile analysis, hardness and crispness are key parameters for evaluating the mechanical properties of cookies, reflecting resistance to deformation and propensity to fracture under stress, respectively [60]. As shown in Table 2, as the concentrations of EGCG and ESLs increase, the hardness of the cookies gradually rises, whereas the crispness notably declines at a concentration of 0.1%. The observed increase in hardness is attributed to the cross-linking between polyphenols and gluten proteins, which strengthens the protein network [61]. The decrease in crispness suggests a reduced propensity for brittle fracture of cookies under stress, which is consistent with the prior finding that high-dose tea polyphenols reduce crispness in baked products [62]. Although these changes are statistically significant, their magnitude is relatively small. Regarding color, in terms of color, the L*, a*, and b* values of cookies changed with increasing EGCG addition. When EGCG was added at 0.05% or higher, L* decreased from 55.89 to 50.58 (decreased by 9.50%), indicating darker color, which is attributed to the formation of brownish polymers via EGCG oxidative polymerization [63,64]. The a* value also decreased from 13.61 to 11.60 (decreased by 14.77%), suggesting that EGCG may inhibit the Maillard reaction [65], thereby reducing the generation of red pigments. However, the b* value exhibited a non-monotonic trend. This may be explained by two possible mechanisms. At low EGCG levels, oxidative polymerization could generate yellow pigments, potentially leading to an increase in b* [66]. At higher EGCG concentrations, the oxidation of EGCG initially produces yellow-colored compounds such as theaflavins; however, as oxidation proceeds, these yellow substances are further oxidized and polymerized into larger and more complex brownish polymers (e.g., thearubigins and the abrownins) [67], and this transition from yellow to brownish products reduces the b value. The ESL treatment showed similar color trends, albeit with slightly different L*, a*, and b* values at each addition level, likely due to the physical barrier of liposomes that partially retarded EGCG oxidation [68].

In combination with the aforementioned results, the inhibitory effects of EGCG and ESLs on CML and CEL were optimal at a concentration of 0.05%, at which the changes in texture and color were relatively minor. hardness increased only slightly compared with the control, crispness did not decrease significantly, and color parameters remained within acceptable ranges. When the concentration increased to 0.1% or higher, the inhibitory effect on AGEs diminished, accompanied by pronounced changed in texture and color.

## 4. Conclusions

In this study, stigmasterol was successfully employed as a cholesterol substitute to formulate EGCG-loaded liposomes (ESLs). Following formulation optimization, ESLs exhibited a high encapsulation efficiency of 92.86%. ESLs markedly enhanced the stability, antioxidant activity, and reactive dicarbonyl compound trapping capacity of EGCG. Furthermore, ESLs demonstrated significantly greater inhibition of two major AGEs, namely CML and CEL, in both chemical model systems and thermally processed cookies. Evaluation of texture and color revealed only minor alterations. Collectively, ESLs represent a promising and practical strategy to stabilize EGCG and concurrently inhibit AGEs formation, thereby expanding its applicability in thermally processed food systems.

## Figures and Tables

**Table 1:** Effect of liposome composition on particle size, zeta potential, and encapsulation efficiency (EE) of EGCG-loaded liposomes. Different letters (*p* < 0.05) indicate significant differences among liposomes of different compositions.

EGCG-Load Liposomes	Particle Size (nm)	Polydispersity Index	Zeta Potential (mv)	EE (%)
EGCG:lecithin				
1:10	156.51 ± 5.98^e^	0.364 ± 0.020^a^	−29.51 ± 1.72 ^a^	66.66 ± 4.21 ^d^
EGCG:cholesterol:lecithin				
1:5:10	234.51 ± 11.94^d^	0.292 ± 0.025 ^b^	−53.95 ± 1.80 ^e^	90.83 ± 2.10 ^a^
EGCG:stigmasterol:lecithin				
1:1:10	152.06 ± 6.55 ^e^	0.257 ± 0.023 ^c^	−45.33 ± 3.54 ^c^	89.96 ± 5.93 ^a^
1:3:10	163.99 ± 7.85 ^e^	0.270 ± 0.011 ^bc^	−50.08 ± 4.44 ^d^	91.86 ± 3.16 ^a^
1:5:10	239.87 ± 16.26 ^d^	0.290 ± 0.020 ^b^	−53.09 ± 0.91 ^e^	92.86 ± 3.16 ^a^
1:7:10	365.23 ± 6.50 ^c^	0.326 ± 0.011 ^ab^	−46.24 ± 6.85 ^c^	85.59 ± 4.64 ^b^
1:5:5	143.38 ± 7.97 ^f^	0.300 ± 0.018 ^b^	−42.33 ± 3.45 ^b^	76.96 ± 3.27 ^c^
1:5:15	524.03 ± 5.09 ^a^	0.366 ± 0.014 ^a^	−42.50 ± 2.58 ^b^	88.30 ± 4.18 ^a^
1:5:20	484.46 ± 8.46 ^b^	0.359 ± 0.012 ^a^	−41.14 ± 0.93 ^b^	79.37 ± 1.29 ^c^

**Table 2:** The texture and color of the cookies.

Texture				Color	
	Hardness (N)	Crispness (N)	L*	a*	b*
Control	18.14 ± 1.43 ^Cd^	16.31 ± 0.98 ^Aa^	55.89 ± 0.51 ^Aa^	13.61 ± 0.05 ^Aa^	25.17 ± 0.57 ^Bb^
EGCG					
0.01%	18.31 ± 1.15^C^	16.44 ± 1.06 ^A^	55.62 ± 0.92 ^A^	13.29 ± 0.41 ^A^	25.17 ± 0.57 ^B^
0.025%	19.92 ± 0.75 ^BC^	16.70 ± 0.65 ^A^	55.77 ± 0.47 ^A^	13.51 ± 0.23 ^A^	28.3 ± 0.22 ^A^
0.05%	21.28 ± 0.61 ^B^	17.14 ± 1.76 ^A^	54.27 ± 0.88 ^B^	12.20 ± 0.27 ^B^	27.45 ± 0.77 ^A^
0.1%	25.08 ± 0.47 ^A^	13.66 ± 0.63 ^B^	50.58 ± 0.66 ^C^	11.60 ± 0.34 ^C^	23.53 ± 0.97 ^C^
ESL					
0.01%	17.54 ± 0.66 ^d^	16.26 ± 0.98 ^a^	55.86 ± 0.62 ^a^	13.04 ± 0.46 ^ab^	25.83 ± 0.12 ^b^
0.025%	20.17 ± 0.63 ^b^	16.22 ± 0.87 ^a^	55.72 ± 0.43 ^a^	13.23 ± 0.6 ^ab^	28.09 ± 0.50 ^a^
0.05%	21.41 ± 0.82 ^b^	17.06 ± 0.25^a^	54.08 ± 1.42 ^b^	12.61 ± 0.05 ^b^	28.37 ± 0.63 ^a^
0.1%	23.59 ± 1.19 ^a^	12.66 ± 1.60 ^c^	50.64 ± 0.58 ^c^	11.21 ± 0.89 ^c^	22.89 ± 0.40 ^c^

Data are presented as mean ± SD. In each column, different uppercase letters (A, B, C) indicate significant differences among the control and EGCG groups at different concentrations (0.01%, 0.025%, 0.05%, 0.1%) (*p* < 0.05). Different lowercase letters (a, b, c, …) indicate significant differences among the control and ESL groups at different concentrations (0.01%, 0.025%, 0.05%, 0.1%) (*p* < 0.05). Uppercase and lowercase letters are compared independently.

## Data Availability

The data presented in this study are available on request from the corresponding author.

## Supplementary Materials

The following supporting information can be downloaded at https://www.mdpi.com/article/10.3390/foods15111997/s1, Figure S1: The chemical structure diagrams of EGCG, cholesterol and stigmasterol.
