# Supplementary figures and images for: Stigmasterol-Based EGCG Liposomes Reduce Nε-(carboxymethyl)lysine (CML) and Nε-(carboxyethyl)lysine (CEL) in a Model System and Cookies
Source: Foods. 2026 Jun 3;15(11):1997. doi: 10.3390/foods15111997 (PMC13256398; doi:10.3390/foods15111997)

Figure S1

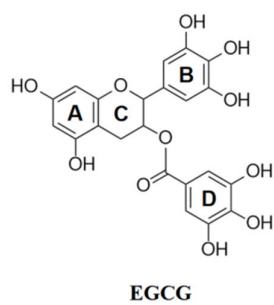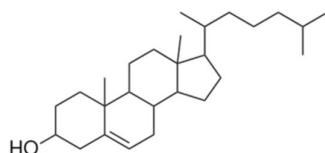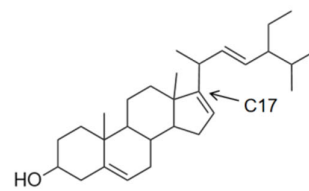

Supplement: Supplementary file 1 [file foods-15-01997-s001.zip › foods-4299572-supplementary.pdf]
